# The time cost of saving money: detouring and connecting time losses in the virtually interlined European airport network

**DOI:** 10.1186/s12544-022-00551-4

**Published:** 2022-06-21

**Authors:** Sarah Meire, Ben Derudder

**Affiliations:** 1 Department of Geography, Ghent University, Campus Sterre, Krijgslaan 281, Building S8, 9000 Ghent, Belgium; 2 KU Leuven Public Governance Institute, KU Leuven, Leuven, Belgium

**Keywords:** European airport network, Virtual interlining, Self-connecting, Connecting time, Detour factor

## Abstract

Virtual interlining, the use of actively marketed self-connecting flight itineraries, is often assumed to be a money-saving air travel strategy. Earlier research on this topic broadly confirmed the money-saving character of virtual interlining, but to date non-monetary costs associated with this price advantage have not yet been systematically examined. In this paper, we address this lacuna by juxtaposing the price advantage of virtual interlining with the potential time costs for the case of indirect flight itineraries in the European airport network. Focusing on those markets where the cheapest virtually interlined itinerary renders a price advantage over its indirect traditional counterpart, we analyse the time cost from two complementary perspectives: (1) connecting time and (2) detour factor. To this end, we query Kiwi.com’s Tequila platform to obtain data on all available flight itineraries in the first week of August, October and December 2019. Based on a series of sign tests, we reveal the time costs of saving money: while virtually interlined itineraries render a price advantage compared to their indirect traditional counterparts, they come with a significantly larger connecting time and detour factor. We reflect on possible explanations, and highlight a number of avenues for future research.

**Supplementary Information:**

The online version contains supplementary material available at 10.1186/s12544-022-00551-4.

## Introduction

In recent years, online travel agencies (OTAs) have started to capitalise on the growing market of self-connecting air travel by constructing and selling self-connecting flight itineraries. These actively marketed self-connecting itineraries are often referred to as *virtually interlined* flights (see, for example, [19, 24, 38, 39]). Although the literature on virtual interlining is still limited in size and scope, earlier research on fares for the European air transport market [33] broadly confirmed, but also to some degree nuanced the assumed money-saving character of virtual interlined air travel. First, comparing the cheapest virtually interlined with *direct* traditional flight itineraries produced mixed results. However, since direct flights are bound to be significantly faster than virtually interlined flights given detouring and connecting times, competition can be assumed to be limited. Second, virtually interlined itineraries were often found to be significantly cheaper than *indirect* traditional itineraries. However, in this case it remains unclear whether detouring and connecting times may offset the money-saving character of virtual interlining. Against this background, focusing on indirect air travel in the European air transport market and in particular those markets where virtual interlining renders a price advantage, this paper extends earlier research by means of an analysis of its (possible) time costs. More specifically, we aim to enhance our understanding of potential differences between virtually interlined itineraries and indirect traditional itineraries from two complementary perspectives: (1) connecting time, and (2) detouring.

The remainder of this paper is organised as follows. In Sect. 2, we elaborate on the virtual interlining concept and situate it within the broader air transport research literature. Based on the review of the literature, we put forward hypotheses regarding the time differences between both types of flights in Sect. 3. Following this, Sect. 4 outlines the data collection process and methodology that allows testing these hypotheses. The results are presented in Sect. 5, followed by a brief discussion and as well as reflections on a possible future research agenda in Sect. 6. The paper is concluded with some concluding remarks in Sect. 7.

## Virtual interlining: rethinking air transport networks?

According to the International Air Transport Association (IATA), *interlining* is “a broad term used to describe one airline selling an itinerary to a customer that involves services provided by another airline” [24, p. 6]. Many commercial agreements encompassing (some form of) interlining relationships exist, including individual interline agreements, codeshare agreements, joint ventures, alliances, and IATA’s Multilateral Interline Traffic Agreements (MITA) [24]. In recent years, however, new interlining models have emerged. While the abovementioned forms of *interlining* (henceforth: *traditional interlining*) encompass a formal pre-agreement by the airline(s), *virtual interlining* is facilitated by a third party without pre-agreement of the airline(s) involved [16]. Virtually interlined flight itineraries thus include (1) one or more off-line connections (i.e., connections involving a change of airlines) between flights operated by unrelated/non-partner carriers, and/or (2) one or more on-line connections (i.e., connections without a change of airlines, see for example [28]) that are not explicitly arranged or facilitated by the airline itself. The latter usually pertains to low-cost carriers (LCCs), although some LCCs are increasingly diverting from the typical LCC business model by providing on-line connections (see, for example, [18, 27, 28]). Consequently, carriers may not be aware of the passengers’ full travel itinerary [24]. Most often, virtually interlined flight itineraries are provided by specialised OTAs via a single transaction [19, 24]. In essence, these online platforms are capitalising on the growing self-connection market by constructing, selling, and—in most cases—insuring seamless flight trajectories not coordinated by airlines themselves. While self-connecting passengers are constructing these trajectories themselves—a process presumably subjected to “uncertainty, volatility and financial risk” [38]—virtual interlined passengers make use of a third party (e.g. an OTA) for constructing and purchasing these. In other words, the main difference with the broader concept of self-connectivity lies in the fact that virtual interlining refers to an *organised* process [39]. Nonetheless, “no strict guidelines exist yet as to the exact features of the virtual interlined product” [39, p. 1]. It is for example still unclear whether and to what extent various forms of *assisted* self-connections (e.g. via airport-led transfer schemes or through airline-facilitated platforms such as ‘Worldwide by easyJet’, see for example [37]) can be categorised as virtual interlining. As a consequence, to date, no consensus exists on a clear-cut boundary between assisted self-connectivity and virtual interlining.

While the literature on self-connectivity is growing (see, for example, [9–11, 17, 18, 27, 31, 32, 37, 43–46, 48]), research on virtual interlining is still limited in size and scope. Nevertheless, given that virtual interlining exhibits some obvious parallels with self-connectivity, insights from the latter literature can inform our understanding of the former. One such relevant insight is that it is estimated that intraregional markets hold the largest potential for self-connectivity [46]. Whereas Barcelona, London Gatwick, London Stansted, Dublin, Oslo, Rome Fiumicino and Dusseldorf airports were found to have the largest (LCC) self-connecting transfer potential [31], destination airports situated in Northern Africa, as well as the Greek, Italian, and Spanish islands exhibit the largest self-connecting potential in terms of reaching previously unserved markets [46]. With respect to the scope of self-connectivity in global air transport markets, Suau-Sanchez et al. [43] found that 4% of current passenger bookings involve a self-transfer. They show that that this figure may increase to 7% and 15%, respectively through the development of airport-facilitated self-connectivity platforms and the full incorporation of self-connecting flight itineraries in all booking platforms. In this context, the fuzzy border between assisted self-connectivity and virtual interlining becomes particularly relevant.

In contrast to the limited academic literature on virtual interlining, there is a considerable body of non-academic literature on the topic. This literature points to an increasing number of virtual interlining travel distributors (see, for example, [38]). This is evidenced by numerous recent platforms, including TripStack, Kiwi.com, Airsiders, and Dohop. According to Boguslawski [3], business development executive and co-founder at TripStack, virtual interlining “has grown from a niche product offered by a few online players to a viable travel option during the Covid-19 pandemic” (see also [25]). Moreover, it is expected that virtual interlining will gain further popularity. According to TripStack, for example, virtually interlined itineraries may ultimately capture up to 15% of all bookings [12], an estimate that corresponds with the estimate of the overall potential of self-connectivity put forward in Suau-Sanchez et al. [43]. More recently, a focus on *multimodal* virtual interlining has emerged (see [12]), facilitating seamless connections between (high-speed) rail and air travel.

The network that is created through virtual interlined connections may benefit airlines, airports, as well as the travelling public. Airlines, for example, may benefit from a wider customer base and increased economies of density [12, 38]. In a similar vein, airports may obtain additional non-aeronautical revenues (see also [44]). Virtual interlined passengers, in turn, generally benefit from a lower monetary trip cost relative to the cheapest indirect traditional flight alternative [33]. As is the case with the concept of self-connectivity, however, there are also several challenges associated with virtual interlining. First, passengers risk missing a virtual interlined connection at their own expense, unless the transfer is insured/guaranteed by the party providing the virtually interlined flight ticket. Second, in most cases, passengers still have to recheck their baggage and go through immigration at every transfer airport. In our empirical analysis, however, the latter is less relevant given our focus on intra-European air travel: only in a minority of cases, a transfer airport is situated outside the Schengen Area.

In this paper, we aim to contribute to this body of literature by arguing that evaluations of virtual interlining should move beyond mere cost-effectiveness. This implies adopting a more comprehensive ‘itinerary choice problem’ approach (see [20]), which includes examining multiple non-monetary costs accompanying the price advantage of virtual interlining. Although circa 60% of online leisure travellers end up purchasing the flight with the lowest fare [22, 41, in 20], air travellers also generally make trade-offs when choosing among different itineraries. This implies taking into account a range of factors including fare levels, scheduling convenience, and frequent flyer programs (see, for example, [36]). As Adler et al. [1, p. 26] state: “Although fare differences are clearly important, airlines, airports, and other service providers can offset even large fare differences with a variety of service features”. In sum, service variables, individual characteristics, as well as trip context may significantly affect passengers’ choice for an itinerary [1]. In this paper, we focus on an important element of these non-monetary costs: possible differences in the time it takes to connect two markets. In the next section, we put forward hypotheses regarding the possible differences in the two chief dimensions of non-monetary costs: detouring and connecting time.

## A bittersweet travel product? Hypotheses on the possible time costs

**Fig. 1:**
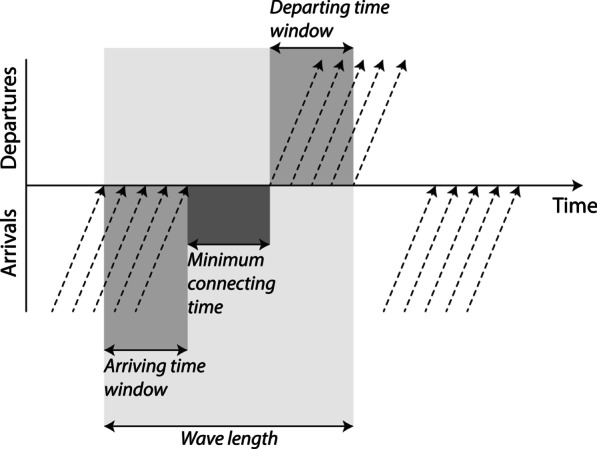
Theoretical configuration of a wave-system structure (adapted from [15, 35])

We hypothesise that traditional flight itineraries will generally outperform their virtual interlined counterparts in terms of connecting time. In contrast to the network shaped by traditional connections, there is no time-based coordination of connections within the virtually interlined flight network. As argued by Grimme [21, p. 4] in a study on LCC self-connections, non-traditional connections are mostly generated “by chance and not systematically”. Indeed, in a virtually interlined flight network, there is no wave-system structure (see, for example, [4, 5, 13, 34], for more information on this concept) to maximise the potential for connectivity whilst minimising connecting times (Fig. 1). Therefore, virtually interlined flight schedules can be assumed to entail longer connecting times. At the same time, however, focusing on self-connectivity in the European air transport market, Malighetti et al. [32] showed that the majority of *fastest* one-stop indirect connections involve a self-transfer, a finding that may also apply to virtual interlined connections. However, because we focus on the cheapest flight itineraries rather than the fastest, we hypothesize that virtually interlined flights will on average take longer.

The geographical detour factor serves as a proxy for the detour time factor given the many assumptions (e.g. speed estimates) needed to compute the actual in-flight time of a theoretical non-stop flight between the origin and destination airports (see for example [44], for a short reflection on the minor difference between both approaches). The geographical detour factor is a widely used parameter in air transport studies [42], and is defined as the ratio between the total distance of the indirect itinerary and the straight distance between the origin and destination airports. Hence, a detour factor of 1.2 implies that the indirect itinerary covers 20% more distance compared to the theoretical non-stop flight. Similar to the connecting time component, it is indicative of the quality of the connection(s) within the indirect flight trajectory. In contrast to the connecting time component, however, it is not possible to specify an unambiguous hypothesis regarding the difference in geographical detour factor between virtually interlined and traditional flights. Obviously, much will depend upon the spatial configuration of the airlines’ bases, and the location of the connecting node(s) in particular. For example, in a comparison of hub-and-spoke networks and mesh networks with on-line connecting services, Klophaus and Fichert [27] argue that a mesh network “provides additional direct links to avoid routings via the hub”, thus lowering the average geographical detour factor. Based on this, it can be argued that the virtually interlined flight network will comprise a larger number of possible intermediate connection points leading to a lower geographical detour factor. However, as customers often focus on the *cheapest* flight itineraries, there may be a large(r) geographical detour factor due to the inclusion of low(er) demand air transport markets and/or LCC airport bases, possibly entailing more remote airports and/or secondary airports with a more limited airside connectivity.

In the next section, we outline the data and method used to evaluate these possible differences in time costs.

## Data and method

Our analysis encompasses 577 airports within the European air transport market, including the EU28 (still comprising the United Kingdom at the time of data gathering) plus Norway, Switzerland, Iceland, Bosnia and Herzegovina, Montenegro, Serbia, Kosovo, Albania, the Republic of North Macedonia, Belarus, Ukraine, Moldova, the Faroe Islands, Guernsey, Man and Jersey. Following [33], we queried Kiwi.com’s B2B platform Tequila to collect (both virtually interlined as well as traditional) scheduled flight data. To increase the statistical robustness of the analysis and take into account the potential flexibility of travellers towards selecting a departure date, we gathered data on all available one-way flight itineraries departing in the first week of August (high season), October (shoulder season), and December (low season) 2019. Price variations related to the time of booking were to some extent accounted for by conducting three data collection rounds (a, b, and c) for each departure date.

The Tequila Search Application Programming Interface (API) was implemented in a Python script, and provided with the following query parameters: the departure and arrival airports, the departure date, a maximum of three flight transfers and one adult passenger. The ‘guarantee’ parameter of the API’s response body—which is provided for each flight *segment*—equals ‘false’ (1) if the airline covers the layover (between the previous flight segment and the flight segment under consideration), (2) in case there is no layover at all (i.e. when a direct flight trajectory is presented, after which the flight will not be taken into accou nt in the analysis), or (3) in case the respective flight segment is the first segment of the itinerary, and therefore not provided with a guarantee to make the connection. In contrast, the ‘guarantee’ parameter equals ‘true’ if Kiwi.com provides (and insures) the connection instead of the airline. Consequently, we denote all itineraries for which there is at least one connection with a Kiwi.com guarantee as a virtually interlined flight trajectory.

In the next step, all relevant data is extracted (or computed) from the API’s response bodies. An overview of the markets where the virtually interlined itinerary has a price advantage is provided in Additional file 1: Appendix A (Table A.1). The total transfer times are extracted by summing the differences between the segments’ UTC arrival time and the connecting segments’ UTC departure time. To compute the geographical detour factor, we (1) calculated the great-circle distance (GCD) of the connecting flight itineraries based on the coordinates of the origin, destination, and transfer airports and (2) the GCD of a hypothetical non-stop flight between the origin and destination airports. The geographical detour factor is then calculated by taking the ratio of both values:

1 $$GeoDetour = \frac{GCD\,of\,the\,connecting\,itinerary}{{GCD\,of\,a\,hypothetical\,nonstop\,flight\, between\, the\, OD\, airports}}$$

**Fig. 2:**
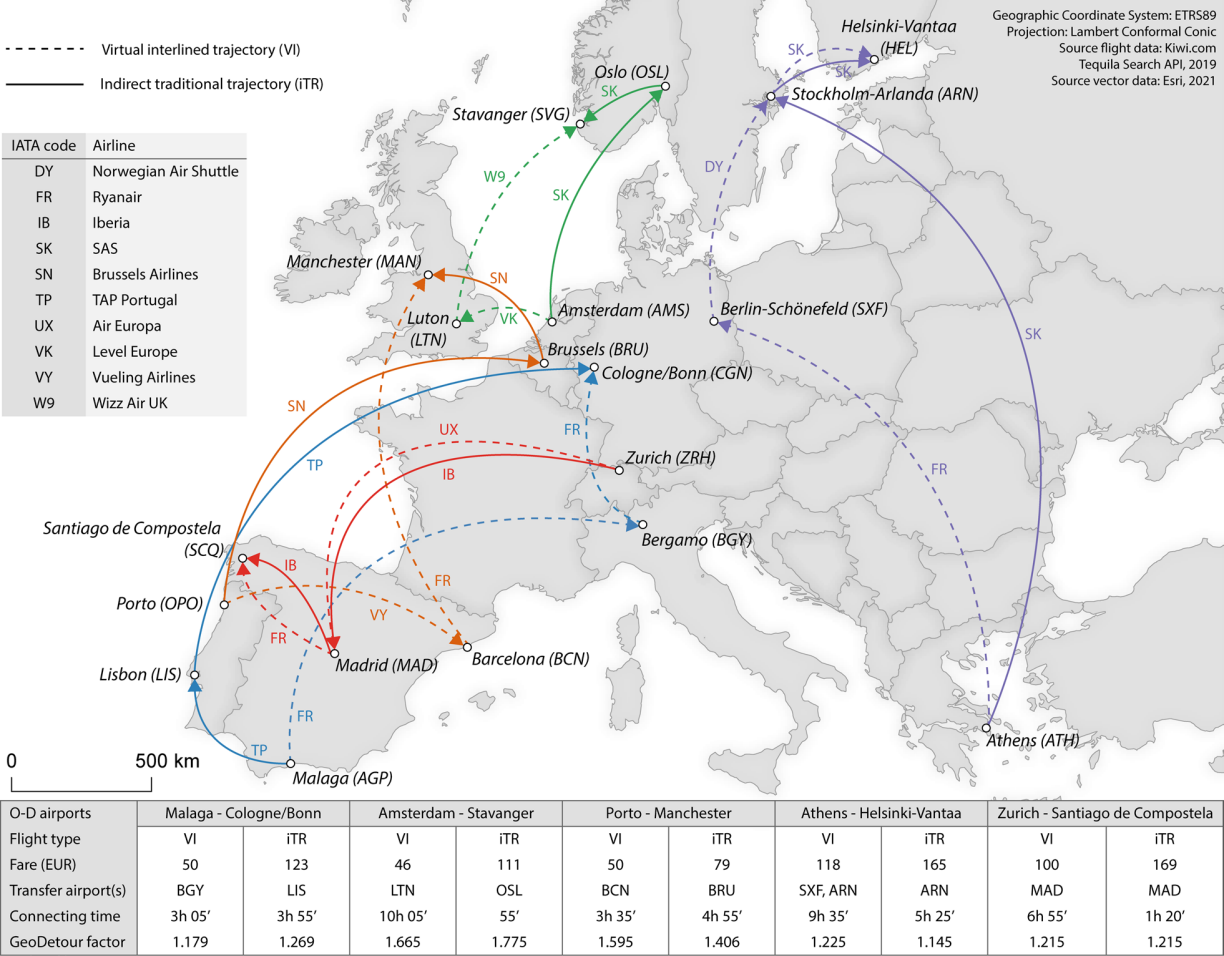
Dataset examples (departure on 02 October 2019, data collection round b). The connection lines are for illustrative purposes only, and therefore do not correspond to the great circle path

Figure 2 illustrates five relevant examples from the dataset (departure on 02 October 2019, data collection round b). The cheapest virtually interlined flight between Malaga and Cologne/Bonn airport consists of two Ryanair flights and has a price advantage of 73 euros. Moreover, it has a smaller connecting time (3 h and 5 min instead of 3 h and 55 min) as well as a smaller geographical detour factor (1.179 instead of 1.269) compared to the traditional itinerary operated by TAP. The Athens-Helsinki market shows the obverse patterns. In this market, passengers travelling on the virtually interlined flight (118 euros) have to spend an additional 4 h and 10 min in transit relative to the traditional alternative (165 euros). In addition, it has a larger geographical detour factor (1.225 instead of 1.145). In this case, the price advantage translates into a higher overall time cost.

The following examples in turn demonstrate the complementarity between connecting and detouring time. The virtually interlined flight between Amsterdam and Stavanger combines a Level Europe^1^ and a Wizz Air UK flight, and has a price advantage of 65 euros. However, this cheaper price comes at a high cost in terms of connecting time. Whereas the traditional itinerary includes a transfer of 55 min, passengers have to wait in transit for circa 10 h when travelling on the virtually interlined flight schedule. In contrast to the connecting time component, however, the traditional itinerary has a higher detouring cost (1.775 instead of 1.665).

The opposite pattern is observed in the Porto-Manchester market. For this market, the virtually interlined flight (50 euros) combining a Vueling Airlines and a Ryanair flight involves a transfer at Barcelona airport, generating a geographical detour factor of 1.595. The traditional itinerary (79 euros) encompasses two Brussels Airlines flights with a transfer at Brussels Airport, implying a smaller geographical detour factor (1.406). However, in this case the traditional itinerary entails a longer connecting time (4 h and 55 min instead of 3 h and 35 min). In some cases, there is no difference in connecting and/or detouring time between both types of flights. In the Zurich-Santiago de Compostela market, for example, both the virtually interlined and the traditional flight encompass a transfer at Madrid, generating an identical geographical detour factor (1.215). There is, however, a large difference in detouring cost. Whereas the traditional itinerary operated by Iberia involves a transfer of 1 h and 20 min, the virtually interlined flight combining an Air Europa and a Ryanair flight involves a transfer of 6 h and 55 min. Taken together, numerous diverging patterns can be found, illustrating the need for a formal analysis of the difference distributions.

Given that the connecting time difference distributions (i.e., iTR-VI connecting time) do not follow a normal distribution (as indicated by a Kolmogorov–Smirnov test, D(8822–25,079) = 0.036–0.075, *p* < 0.001)^2^ and are overall moderately skewed (S_k_ = between − 0.803 and − 0.350), a series of sign tests are performed to (1) assess whether there is a statistically significant difference in connecting time, and (2) to determine the direction in which this potential difference is manifested. For a detailed description on how to compute and interpret the sign test, we refer to Hollander et al. [23]. The null hypothesis states that the median of connecting time differences equals zero, indicating that there is no difference between both types of flight in terms of connecting time. Similarly, the difference distributions of the geographical detour factor are not normally distributed (i.e., D(8822–25,079) = 0.190–0.278, *p* < 0.001) nor symmetrically shaped (S_k_ = between − 29.039 and 12.647). Hence, another a series of sign tests was conducted. An overview of the descriptive statistics of the difference distributions, as well as the sign test results, are provided in Additional file 1: Appendix B (Tables B.1 to B.6) and Additional file 1: Appendix C (Tables C.1 to C.6).

## Results

### Connecting time

**Fig. 3:**
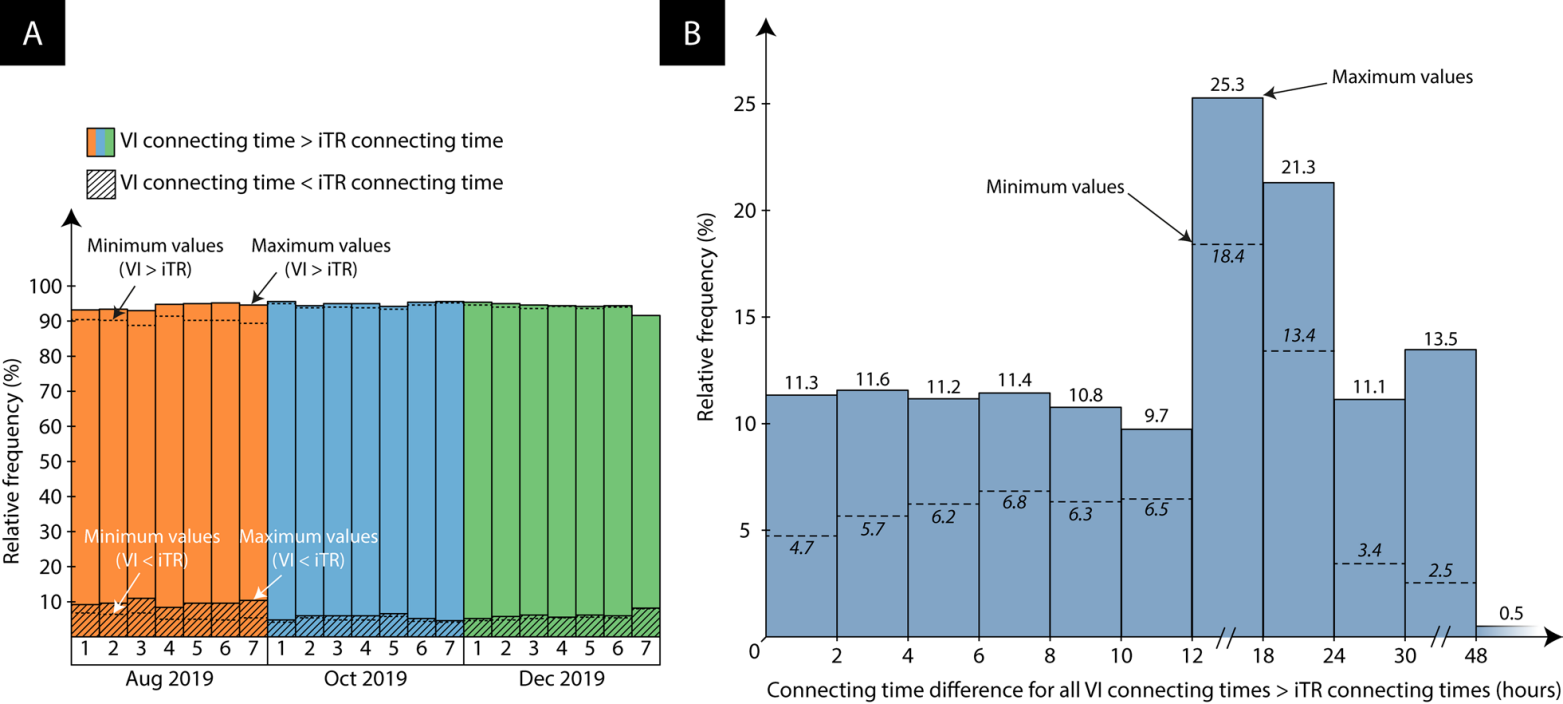
Connecting time differences

For all departure dates and data collection rounds, a statistically significant difference in connecting time is observed (Asymptotic Sig. (2-sided test) < 0.001), with most traditional itineraries rendering shorter connecting times. During the first week of August 2019, for example, the traditional itineraries have shorter connecting times in 88.8–95.1% of all markets (i.e., the minimum and maximum negative iTR-VI differences across all 21 departure dates and data collection rounds, see also Fig. 3A. However, the opposite situation is also sometimes found: for 4.8–11% of the airport pairs (i.e., the minimum and maximum positive iTR-VI difference across all 21 departure dates and data collection rounds), the virtually interlined flight renders a shorter connecting time. Only in a minority of cases (0.1–0.2%), both types of flight exhibit an identical connecting time. Similar results are obtained with respect to the departure dates in the first week of October 2019: for 93.3–95.7% of the airport pairs, the traditional flight itinerary has a longer connecting time. In only 4.2–6.6% of cases, the opposite situation is observed. With respect to the departure dates in the first week of December 2019, these two categories respectively encompass 91.5–95.3% and 4.6–8.3% of the airport pairs.

Figure 3B, in turn, shows the relative frequency of the magnitude of the connecting time differences for all markets where the virtually interlined flight renders a *longer* connecting time (i.e., VI connecting time > iTR connecting time). Cumulatively, for 67–83.7% of the airport pairs, the connecting time difference exceeds 6 h. For 39.8–62.2% of the airport pairs, the virtual interlined passengers spend more than an additional 12 h at the transfer airport(s) relative to those travelling on the traditional schedule. While 6.1–24.6% of the connecting time differences even exceeds 24 h, travellers rarely (0–0.5%) lose more than 48 h at the transfer airport(s) by choosing the virtually interlined flight option.

**Fig. 4:**
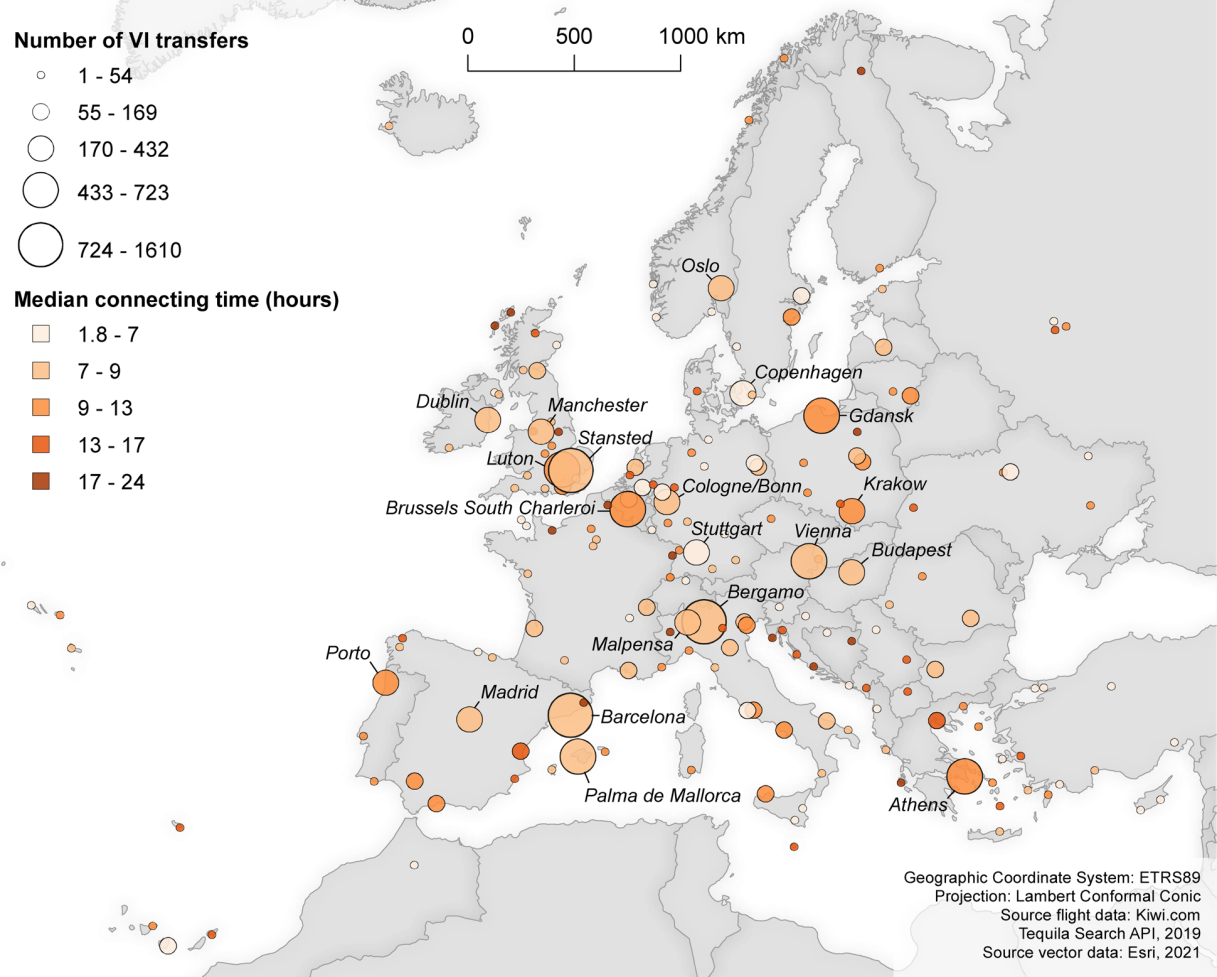
Connecting within the virtually interlined flight network (02 October 2019, data collection round b)

In order to reflect in more detail upon the connecting times as well as the spatial configuration of the transfer airports in the virtually interlined flight network, Fig. 4 visualises (1) the number of times an airport acts as transfer airport within the virtually interlined schedules, and (2) the median connecting time for each transfer airport (departure date: 02 October 2019; data collection round: b).

The top-10 transfer airports are Barcelona, London Stansted, Milan Bergamo, London Luton, Athens, Palma de Mallorca, Brussels South Charleroi, Gdansk, Vienna, and Dublin Airport. Overall, it can be observed that virtual interlined transfers predominantly take place at major LCC airport bases. This is in line with the findings by [33], who showed that virtually interlined flights rendering a price advantage are mostly operated by Europe’s leading LCCs. Barcelona Airport, for example, ranks first in the top-10 transfer airports and is the primary airport base of Vueling Airlines [8]. London Stansted, in turn, ranks second and constitutes Ryanair’s largest airport base [40]. Finally, Milan-Bergamo completes the top-3 and forms the third largest airport base of Ryanair. It can furthermore be observed that multiple secondary airports stand out, presumably due to (ultra-)LCCs which mostly operate on low-density routes between secondary airports (see, for example, [14]). An example hereof is Brussels South Charleroi Airport, which constitutes the sixth largest base of Ryanair [40]. With respect to connecting times, the top-10 transfer airports exhibit median connecting times ranging from circa 10 h (Dublin Airport) to circa 12 h (Gdansk Airport).

**Fig. 5:**
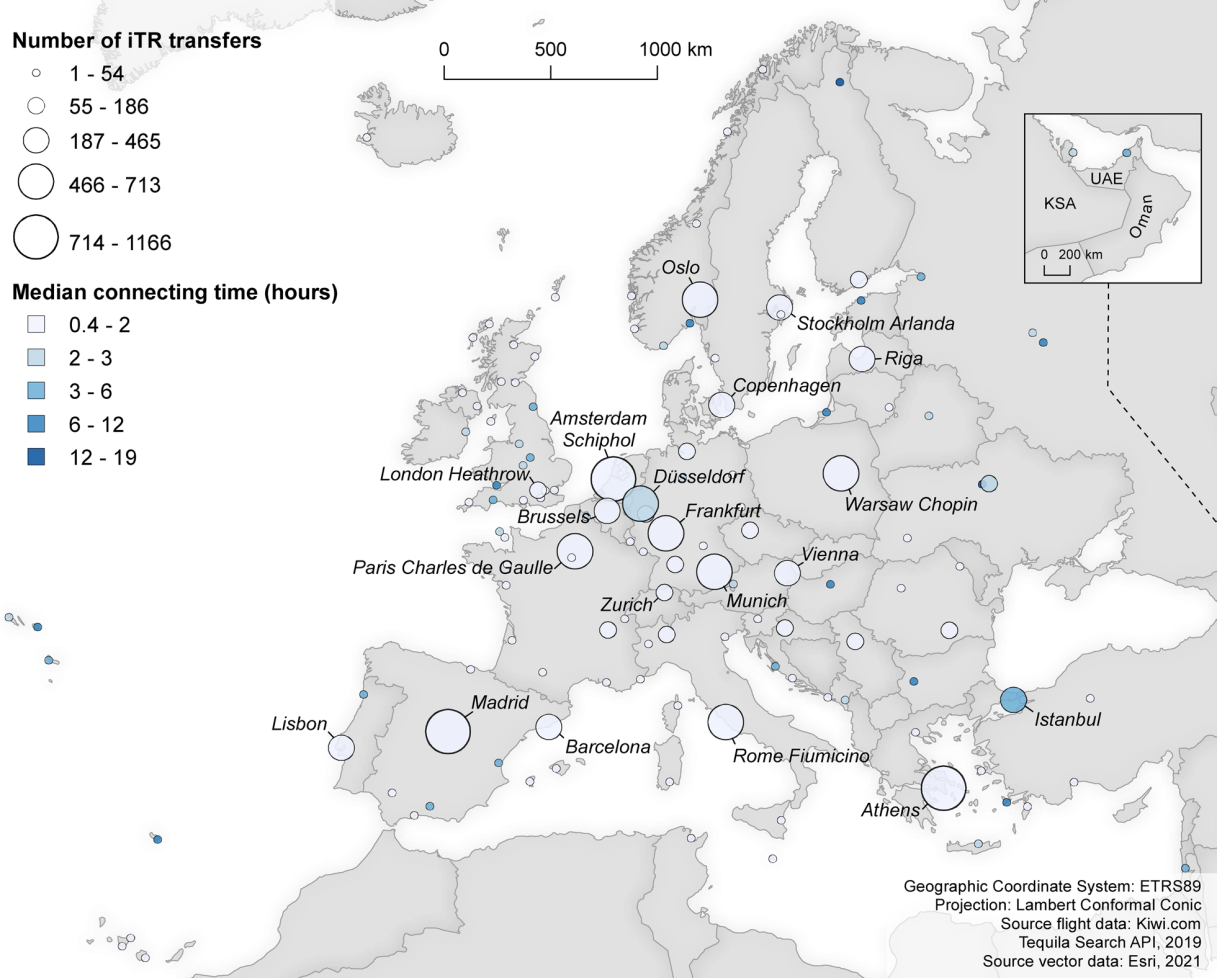
Connecting in the traditional flight network (02 October 2019, data collection round b)

In parallel to Figs. 4,  5 visualises (1) the number of times an airport acts as transfer airport within the traditional schedules, and (2) the median connecting time for each transfer airport (departure date: 02 October 2019; data collection round: b).

In the traditional flight network, Europe’s leading full service network carriers’ (FSNC) hub airports are dominant. In this case, the top-10 transfer airports are Amsterdam Schiphol, Athens, Madrid, Rome Fiumicino, Oslo, Paris Charles de Gaulle, Munich, Frankfurt, Düsseldorf, and Warsaw Airport. Many of these airports constitute a primary base of a FSNC. Amsterdam Schiphol, for example, ranks first in the top-10 and forms the home base of KLM [26]. Frankfurt Airport, in turn, ranks eighth and is the largest Lufthansa hub [30]. Median connecting times associated with these top-10 transfer airports are between approximately one (Munich Airport) and two hours (Düsseldorf Airport), which yet again illustrates the large connecting time differences between both types of networks.

Finally, in order to assess whether there exists a correlation between the (magnitude of) the *positive* fare differences (iTR-VI fare > 0) and the *negative* connecting time differences (iTR-VI travel time < 0), a Kendall’s Tau-b correlation was computed. The Kolmogorov–Smirnov test results and the Kendall’s Tau-b test results are provided in Additional file 1: Appendix D and Additional file 1: Appendix E, respectively. For all departure dates and data collection rounds a weak to very weak negative correlation was found between the positive fare differences and the negative connecting time differences (N = between 8089 and 22,615, *τ*_*b*_ = between − 0.161 and − 0.047, *p* < 0.01). Hence, although the correlation is weak at best, there is some evidence that the greater the price advantage of a virtually interlined flight, the greater the connecting time cost relative to its traditional alternative. This in turn informs research on passengers’ value of time, which will be elaborated on in Sect. 7.

### Geographical detour factor

**Fig. 6:**
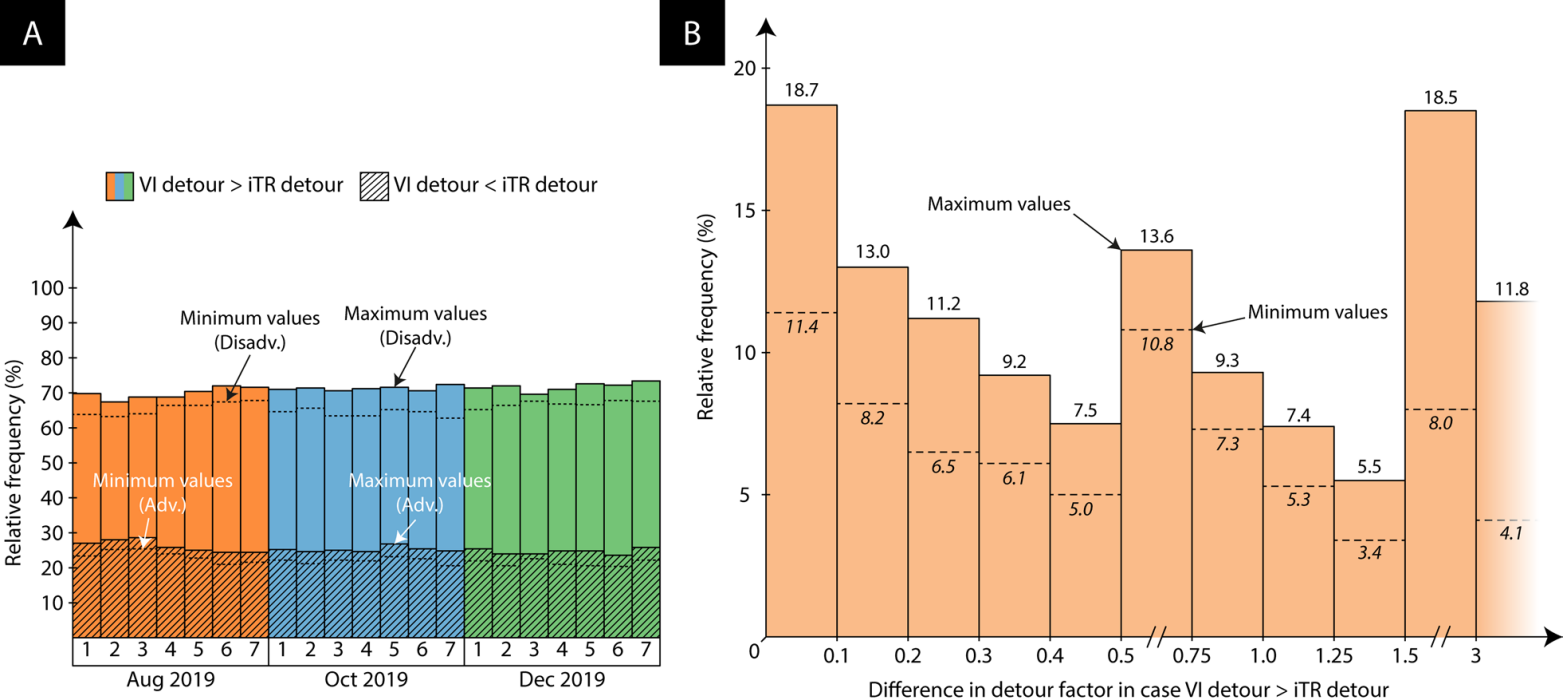
Geographical detour factor differences

For all departure dates and data collection rounds, a statistically significant difference in geographical detour factor is observed (*p* < 0.001), with most traditional itineraries characterised by shorter detours. More specifically, in the first week of August 2019, the traditional itineraries have smaller geographical detour factors in 63.2–72.0% of the markets (see also Fig. 6A). The opposite is observed for 21.1–28.6% of the airport pairs. Similarly, for the first week of October and December 2019, the indirect traditional flight itineraries have smaller geographical detour factors in 62.8–72.3% and 65.3–73.5% of cases, respectively. In contrast, for 20.5–26.9% and 20.5–25.8% of the airport pairs, the virtually interlined flight covers less distance in the first week of October and December 2019, respectively. This shows that the larger number of possible connection points within the virtually interlined flight network does generally not translates into a reduced geographical detour factor when the cheapest flight itineraries are considered. Nonetheless, the relative frequency of the positive/negative differences seem somewhat less pronounced compared to the connecting time differences displayed in Fig. 3A.

Figure 6B, in turn, shows the relative frequency of the magnitude of the negative differences in geographical detour factor (i.e., VI geographical detour factor > iTR geographical detour factor). Cumulatively, for 41.5–62.5% of the respective airport pairs, the difference in geographical detour factor is greater than 0.5. This means that the extra distance covered by the virtually interlined flight equals more than half the GCD of the hypothetical non-stop flight between the origin and destination airports. For 21.3–43.1% of airport pairs, the difference in geographical detour factor is even greater than 1, implying that the extra distance covered by the virtually interlined flight is more than the entire GCD of a hypothetical non-stop flight between the origin and destination airports. For 4.1–11.8% of cases, the difference in geographical detour factor is larger than three.

Similar to the previous section, we furthermore test whether a correlation exists between the (magnitude of) the *positive* fare differences (iTR-VI fare > 0) and the *negative* geographical detour factor differences (iTR-VI geographical detour factor < 0). To this end, a Kendall’s Tau-b correlation is again calculated (see Additional file 1: Appendix E). For the majority of departure dates and data collection rounds, a weak to very weak positive correlation was found between the positive fare differences and the negative detour differences (N = between 5960 and 16,888, *τ*_*b*_ = between 0.023 and 0.130, *p* < 0.01). Although the correlation is weak at best, this implies that the greater the price advantage of a virtually interlined flight, the smaller the difference in geographical detour factor. In contrast, for a single configuration (i.e., 07 August 2019 data collection round c), a very weak, negative correlation was found. In three configurations (i.e., 04 August 2019 data collection round c, 05 August 2019 data collection round c, and 06 August 2019 data collection round c), no statistically significant correlation was found. Given these contradictory results, we cannot speak of a clear/unambiguous correlation between the fare profits and the detour costs associated with virtual interlining.

## Discussion

Our results showed that the price advantage of virtual interlining generally comes with a number of costs in terms of detouring and connecting times compared to the cheapest indirect traditional alternatives. Hence, while virtual interlining is often advertised as a strategy for finding lower fares and even better schedules (see, for example, https://virtualinterlining.io), we argue that these benefits come with some caveats.

From a consumer’s perspective, one of the most noticeable and/or influential drawbacks of the virtual interlined product may be the substantial increase in connecting and detouring time associated with its price advantage. It is therefore valuable to confront the observed travel time disadvantages of virtual interlining with passengers’ value of time. Earlier research showed that, on average, leisure and business travellers are willing to pay $31 and $70 per reduced hour of travel time, respectively [1]. In addition, whereas passengers positively value a connecting time of 15 min above the minimum connecting time (MCT) proposed by the airline(s) [29], this positive perception gradually turns negative with increasing connecting times. Given that the connecting time disadvantages exceed 6 h in 67–83.7% of cases, a considerable number of travellers will end up purchasing the more expensive, indirect traditional itinerary. Moreover, in short-haul markets “passengers are particularly averse to multi-stop connections even if the travel time is theoretically competitive” [7, p. 40]. The question thus arises to what extent passengers are willing to wait for a longer time in transit in exchange for a fare reduction. Related to this, Grimme [21, p. 18] argues that the price difference between an assisted LCC connecting itinerary and a traditional FSNC connecting itinerary “must be higher than the sum of the monetised disutility of the ‘non-seamless’ LCC connection and the value of the difference in travel time” for passengers to choose the non-traditional flight option. Future research may thus be directed towards qualitatively analysing passengers’ willingness to virtual interline. Related to this, another avenue for future research pertains to assessing in more depth the possible heterogeneity in the observed travel time costs. Indeed, given that this paper is the first to juxtapose the fare advantage of virtual interlining with its possible costs in terms of travel time, it predominantly discusses the general insights that could be derived from our dataset without exploring the potential sources of heterogeneity. These results may therefore mask underlying patterns (for example, holiday versus business markets, regional variations, etc.), which may be evaluated in future research.

Apart from the observation that virtual interlined travellers must pay a high(er) detouring time cost in exchange for a cheaper fare, the observed detouring difference should also be evaluated within the context of environmental sustainability. Given the larger geographical detours associated with the virtual interlining product, one may expect that this new type of interline model is more environmentally damaging than traditional interline models. However, due to the many (interacting) factors possibly impacting the environmental efficiency of the individual flight legs (e.g. the aircraft types used), obtaining detailed estimates is far from straightforward. Another future research avenue therefore pertains to evaluating the environmental sustainability of virtual interlined air travel.

This paper focused on juxtaposing the fare *advantage* of virtual interlining with its *drawbacks* in terms of travel time costs. However, even though the virtual interlining product is widely advertised as a money-saving air travel strategy, a claim that is both corroborated and nuanced by [33], several non-financial drivers may push travellers towards buying a (possibly more expensive) virtually interlined flight ticket. Travellers may for example choose to virtually interline—regardless of whether or not it constitutes the cheapest flight option—if this enables them to reach their desired destination in a shorter period of time. By restricting our analyses to those markets where the cheapest virtually interlined flight renders a price advantage, we did not gain an insight into the overall travel time difference between both forms of air travel *irrespective* of their fare difference. Future research on the virtual interlining product may therefore possibly benefit from uncoupling these variables to provide a more comprehensive picture of the overall differences in travel time. Related to this, fare levels most often rest upon a combination of cost-based, demand-based, and service-based pricing mechanisms [2]. In other words, several determinants co-shape the pricing of a flight in a particular O-D market, among which hub dominance, the number of carriers in the market, the presence of LCCs, market share (see [47]), as well as myriad service quality attributes (e.g., flight frequency). One could therefore argue that the fare advantages may to some degree be endogenously effectuated *by*, amongst others, higher travel time costs. Importantly, however, airline fares are typically defined for an O-D market [2], which in the context of this paper deserves specific attention. Indeed, whereas a traditional flight ticket pertains to a single O-D market, a virtually interlined flight ticket in fact encompasses multiple O-D markets as it essentially entails a combination of separate flight tickets. This adds another layer of complexity to the question about how virtually interlined and traditional ticket fare levels are being shaped. A possible avenue for future research therefore pertains to identifying the fare determinants that are at play in our flight da tabase and, hence, the key factors effectuating the fare advantage of the virtual interlining travel product.

From a more theoretical perspective, future research may also focus on defining the exact features of the virtual interline product and varying forms of *assisted* self-connecting air travel. This seems particularly relevant given the dynamic nature of air transport networks and airline business models. For example, according to Burghouwt and de Wit [6, p. 109] “market perspectives for the so-called long-haul, low-cost model in Europe may become viable due to technological innovations. Such operations may further develop into low-cost hub-and-spoke systems”. The question thus arises if and to what extent LCCs will actively engage in creating and/or facilitating interline networks themselves, and how this will interact with and impact the virtual interlined product offered by third parties.

## Concluding remarks

In this study, we addressed some of the drawbacks of virtual interlined air travel. Focusing on the markets within which virtual interlining renders a price advantage relative to its indirect traditional counterpart, we examined the time cost differences between both types of flight from two complementary perspectives: (1) detouring and (2) connecting time. Our results clearly showed the time costs of saving money: while the virtually interlined flights render a price advantage, they entail a significantly larger connecting time and detour factor. Hence, future research may be directed towards (1) qualitatively analysing this new interline model within the context of the itinerary choice problem, and in particular with respect to passengers’ value of time, (2) the environmental sustainability of different forms of interline models, and (3) the defining of the virtual interlined product and its interaction with the dynamics of air transport networks and evolving airline business models.

## Supplementary Information

**Additional file 1.** Appendices.

## Data Availability

The raw dataset supporting the conclusions of this article is not publicly available due to access/contractual restrictions. Data are however available from the authors upon reasonable request and with permission of Kiwi.com.

## Abbreviations

OTA: Online travel agency
IATA: The International Air Transport Association
MITA: Multilateral Interline Traffic Agreements
LCC: Low cost carrier
API: Application Programming Interface
GCD: Great-circle distance
FSNC: Full service network carrier
MCT: Minimum-connecting time

## Footnotes

1 Level Europe was still operational at the time of data gathering.

2 The minimum and maximum values across all 21 departure dates and data collection rounds (a, b, and c).
