# Supplementary material for: The time cost of saving money: detouring and connecting time losses in the virtually interlined European airport network
Source: Eur Transp Res Rev. 2022 Jun 21;14(1):26. doi: 10.1186/s12544-022-00551-4 (PMC9209317; doi:10.1186/s12544-022-00551-4)
Supplement: Supplementary file 1 — Additional file 1. Appendices. [file 12544_2022_551_MOESM1_ESM.pdf]

Appendix A: Overview of the air transport markets included in the analysis

Table A.1: Number of markets where the cheapest virtually interlined flight renders a price advantage relative to its *indirect* traditional counterpart.

Departure date	Data collection round		
	A	B	C
01 August 2019	21 532	23 911	24 470
02 August 2019	21 678	22 927	24 376
03 August 2019	21 288	21 814	22 905
04 August 2019	22 568	22 976	24 143
05 August 2019	22 716	23 565	25 079
06 August 2019	22 322	23 463	24 358
07 August 2019	22 039	23 337	22 302
01 October 2019	10 863	12 710	16 416
02 October 2019	10 792	11 639	20 186
03 October 2019	10 465	11 981	20 440
04 October 2019	10 503	12 299	20 224
05 October 2019	9906	11 564	17 886
06 October 2019	10 368	11 612	17 772
07 October 2019	10 371	12 096	21 232
01 December 2019	10 399	11 006	16 369
02 December 2019	10 181	11 434	15 695
03 December 2019	9407	11 794	14 880
04 December 2019	9811	12 672	14 526
05 December 2019	10 555	14 410	15 634
06 December 2019	12 220	15 140	16 347
07 December 2019	8822	12 850	13 747

Appendix B: Descriptive statistics on the transfer time and geographical detour factor difference distributions (for all markets where virtual interlining renders a price advantage)

Table B.1: Descriptive statistics (for August, 2019) on the iTR-VI transfer time difference distribution.

Departure date	Round	Median	Mean	Skewness	Kurtosis	KS-statistic	KS p-value
01 August 2019	A	-11.250	-12.563	-0.606	0.437	0.054	.000
	B	-12.833	-13.891	-0.491	0.151	0.048	.000
	C	-10.583	-11.830	-0.497	0.379	0.049	.000
02 August 2019	A	-11.417	-13.256	-0.592	0.219	0.066	.000
	B	-13.500	-14.930	-0.462	-0.100	0.058	.000
	C	-11.083	-12.419	-0.445	0.199	0.050	.000
03 August 2019	A	-13.250	-14.090	-0.351	0.064	0.040	.000
	B	-14.667	-15.568	-0.350	-0.102	0.041	.000
	C	-11.083	-12.185	-0.423	0.333	0.042	.000
04 August 2019	A	-13.833	-14.945	-0.428	-0.039	0.049	.000
	B	-14.917	-15.831	-0.371	-0.180	0.043	.000
	C	-12.417	-13.389	-0.402	0.157	0.040	.000
05 August 2019	A	-13.500	-14.621	-0.511	0.092	0.046	.000
	B	-14.500	-15.507	-0.414	-0.079	0.041	.000
	C	-11.333	-12.200	-0.366	0.217	0.036	.000
06 August 2019	A	-13.500	-14.537	-0.492	0.045	0.044	.000
	B	-14.750	-15.788	-0.435	-0.135	0.045	.000
	C	-11.167	-12.054	-0.411	0.351	0.036	.000
07 August 2019	A	-12.333	-13.516	-0.514	0.231	0.050	.000
	B	-13.167	-14.303	-0.501	0.121	0.047	.000
	C	-10.417	-11.352	-0.431	0.372	0.040	.000

Table B.2: Descriptive statistics (for October, 2019) on the iTR-VI transfer time difference distribution.

Departure date	Round	Median	Mean	Skewness	Kurtosis	KS-statistic	KS p-value
01 October 2019	A	-9.500	-10.762	-0.735	0.932	0.063	.000
	B	-10.083	-11.234	-0.682	0.814	0.056	.000
	C	-11.083	-12.292	-0.629	0.663	0.054	.000
02 October 2019	A	-8.833	-10.296	-0.704	0.775	0.073	.000
	B	-9.333	-10.904	-0.691	0.585	0.072	.000
	C	-10.750	-12.056	-0.601	0.356	0.059	.000
03 October 2019	A	-9.167	-10.690	-0.803	0.901	0.072	.000
	B	-9.500	-11.274	-0.702	0.571	0.075	.000
	C	-11.750	-13.146	-0.599	0.151	0.059	.000
04 October 2019	A	-10.000	-11.279	-0.640	0.600	0.062	.000
	B	-10.500	-11.743	-0.565	0.538	0.056	.000
	C	-11.917	-12.854	-0.474	0.405	0.043	.000
05 October 2019	A	-9.750	-11.235	-0.731	0.744	0.069	.000
	B	-9.917	-11.528	-0.635	0.623	0.067	.000
	C	-12.167	-13.374	-0.492	0.177	0.053	.000
06 October 2019	A	-10.833	-12.079	-0.655	0.580	0.059	.000
	B	-11.500	-12.544	-0.568	0.343	0.056	.000
	C	-13.583	-14.215	-0.454	0.053	0.041	.000
07 October 2019	A	-9.917	-11.083	-0.768	0.948	0.059	.000
	B	-10.083	-11.396	-0.754	0.890	0.063	.000
	C	-12.917	-14.001	-0.560	0.263	0.046	.000

Table B.3: Descriptive statistics (for December, 2019) on the iTR-VI transfer time difference distribution.

Departure date	Round	Median	Mean	Skewness	Kurtosis	KS-statistic	KS p-value
01 December 2019	A	-11.833	-12.345	-0.546	0.477	0.048	.000
	B	-12.250	-12.585	-0.466	0.468	0.039	.000
	C	-14.583	-14.750	-0.419	0.089	0.043	.000
02 December 2019	A	-10.083	-11.334	-0.684	0.674	0.061	.000
	B	-10.417	-11.418	-0.639	0.641	0.056	.000
	C	-12.833	-13.743	-0.583	0.330	0.046	.000
03 December 2019	A	-10.417	-11.493	-0.594	0.580	0.058	.000
	B	-10.833	-11.947	-0.565	0.372	0.055	.000
	C	-12.167	-12.959	-0.508	0.259	0.048	.000
04 December 2019	A	-9.833	-11.399	-0.775	0.743	0.072	.000
	B	-10.833	-12.163	-0.727	0.583	0.060	.000
	C	-11.417	-12.589	-0.673	0.543	0.057	.000
05 December 2019	A	-11.500	-12.132	-0.513	0.274	0.058	.000
	B	-12.917	-13.425	-0.475	0.078	0.052	.000
	C	-13.083	-13.731	-0.465	0.055	0.047	.000
06 December 2019	A	-10.417	-11.860	-0.762	0.861	0.064	.000
	B	-11.833	-13.099	-0.589	0.301	0.051	.000
	C	-12.583	-13.733	-0.506	0.239	0.045	.000
07 December 2019	A	-9.667	-10.663	-0.546	0.753	0.052	.000
	B	-11.000	-11.844	-0.458	0.396	0.043	.000
	C	-11.500	-12.398	-0.528	0.349	0.043	.000

Table B.4: Descriptive statistics (for August, 2019) on the iTR-VI geographical detour factor difference distribution.

Departure date	Round	Median	Mean	Skewness	Kurtosis	KS-statistic	KS p-value
01 August 2019	A	-0.124	-0.400	12.647	1371.176	0.218	0.000
	B	-0.225	-0.566	-4.913	69.389	0.203	0.000
	C	-0.125	-0.428	-8.892	213.121	0.220	0.000
02 August 2019	A	-0.122	-0.363	-4.870	75.451	0.201	0.000
	B	-0.174	-0.465	-5.958	83.577	0.199	0.000
	C	-0.129	-0.438	-6.783	171.191	0.218	0.000
03 August 2019	A	-0.131	-0.320	-1.923	190.039	0.193	0.000
	B	-0.199	-0.461	-7.138	141.682	0.204	0.000
	C	-0.176	-0.509	-5.088	77.776	0.202	0.000
04 August 2019	A	-0.158	-0.433	-4.594	79.395	0.197	0.000
	B	-0.202	-0.524	-8.894	189.344	0.206	0.000
	C	-0.189	-0.595	-7.645	152.897	0.217	0.000
05 August 2019	A	-0.177	-0.506	-6.710	96.329	0.207	0.000
	B	-0.233	-0.588	-7.803	130.361	0.214	0.000
	C	-0.175	-0.600	-7.587	131.069	0.219	0.000
06 August 2019	A	-0.209	-0.574	-7.012	129.522	0.203	0.000
	B	-0.281	-0.662	-7.398	116.062	0.213	0.000
	C	-0.202	-0.641	-7.974	173.273	0.215	0.000
07 August 2019	A	-0.192	-0.529	9.908	1163.713	0.228	0.000
	B	-0.259	-0.622	-7.741	151.727	0.214	0.000
	C	-0.205	-0.667	-7.159	128.309	0.227	0.000

Table B.5: Descriptive statistics (for October, 2019) on the iTR-VI geographical detour factor difference distribution.

Departure date	Round	Median	Mean	Skewness	Kurtosis	KS-statistic	KS p-value
01 October 2019	A	-0.150	-0.569	-10.390	232.948	0.213	0.000
	B	-0.184	-0.629	-9.006	160.079	0.215	0.000
	C	-0.258	-0.711	-5.720	62.478	0.204	0.000
02 October 2019	A	-0.156	-0.569	-10.000	220.847	0.219	0.000
	B	-0.184	-0.619	-8.560	144.877	0.217	0.000
	C	-0.288	-0.762	-5.338	113.026	0.215	0.000
03 October 2019	A	-0.135	-0.485	-5.532	75.535	0.194	0.000
	B	-0.180	-0.564	-25.305	1374.225	0.244	0.000
	C	-0.251	-0.704	-13.338	416.623	0.225	0.000
04 October 2019	A	-0.126	-0.479	-7.165	147.484	0.208	0.000
	B	-0.193	-0.608	-10.186	209.270	0.214	0.000
	C	-0.260	-0.720	5.387	806.392	0.233	0.000
05 October 2019	A	-0.154	-0.438	-4.401	68.503	0.190	0.000
	B	-0.182	-0.560	-4.988	58.807	0.192	0.000
	C	-0.272	-0.732	-10.280	315.144	0.214	0.000
06 October 2019	A	-0.148	-0.494	-4.162	70.357	0.190	0.000
	B	-0.167	-0.551	-6.514	108.288	0.200	0.000
	C	-0.251	-0.727	-6.173	79.267	0.206	0.000
07 October 2019	A	-0.126	-0.503	-7.301	122.998	0.202	0.000
	B	-0.180	-0.590	-7.784	137.891	0.210	0.000
	C	-0.305	-0.791	-7.161	126.686	0.206	0.000

Table B.6: Descriptive statistics (for December, 2019) on the iTR-VI geographical detour factor difference distribution.

Departure date	Round	Median	Mean	Skewness	Kurtosis	KS-statistic	KS p-value
01 December 2019	A	-0.185	-0.680	-8.962	155.067	0.226	0.000
	B	-0.147	-0.609	-12.007	286.916	0.236	0.000
	C	-0.324	-0.881	-14.928	526.426	0.227	0.000
02 December 2019	A	-0.173	-0.648	-20.965	864.553	0.259	0.000
	B	-0.168	-0.622	-28.656	1383.729	0.268	0.000
	C	-0.317	-0.839	-21.972	937.163	0.247	0.000
03 December 2019	A	-0.173	-0.634	-13.197	331.296	0.248	0.000
	B	-0.212	-0.711	-26.607	1204.942	0.271	0.000
	C	-0.272	-0.816	-23.123	929.816	0.261	0.000
04 December 2019	A	-0.169	-0.617	-29.039	1328.242	0.278	0.000
	B	-0.213	-0.667	-28.245	1453.529	0.256	0.000
	C	-0.307	-0.800	-25.986	1186.392	0.250	0.000
05 December 2019	A	-0.157	-0.619	-22.645	996.224	0.267	0.000
	B	-0.263	-0.778	-22.542	971.865	0.254	0.000
	C	-0.321	-0.825	-18.065	711.093	0.240	0.000
06 December 2019	A	-0.202	-0.718	-18.250	708.991	0.246	0.000
	B	-0.297	-0.860	-23.765	1038.992	0.251	0.000
	C	-0.344	-0.944	-18.039	654.319	0.246	0.000
07 December 2019	A	-0.197	-0.706	-21.684	1010.480	0.244	0.000
	B	-0.310	-0.854	-17.564	790.402	0.215	0.000
	C	-0.389	-0.999	-16.214	613.901	0.230	0.000

Appendix C: Sign test results (for all markets where virtual interlining renders a price advantage)

Table C.1: Sign test results for the transfer time component (for August, 2019).

Departure date	Data collection	S+	S-	Ties	STS
01 August 2019	A	1521	19981	30	-125.883***
	B	1619	22267	25	-133.593***
	C	2265	22145	60	-127.236***
02 August 2019	A	1529	20108	41	-126.299***
	B	1468	21428	31	-131.904***
	C	2348	21977	51	-125.849***
03 August 2019	A	1646	19612	30	-123.216***
	B	1489	20300	25	-127.430***
	C	2523	20336	46	-117.811***
04 August 2019	A	1250	21289	29	-133.471***
	B	1171	21778	27	-136.023***
	C	2043	22059	41	-128.923***
05 August 2019	A	1130	21561	25	-135.626***
	B	1233	22310	22	-137.359***
	C	2425	22615	39	-127.584***
06 August 2019	A	1170	21127	25	-133.644***
	B	1123	22314	26	-138.414***
	C	2325	21982	51	-126.075***
07 August 2019	A	1238	20764	37	-131.632***
	B	1243	22056	38	-136.347***
	C	2334	19938	30	-117.952***

*** $p < .001$

Table C.2: Sign test results for the transfer time component (for October, 2019).

Departure date	Data collection	S+	S-	Ties	STS
01 October 2019	A	466	10382	15	-95.196***
	B	587	12107	16	-102.239***
	C	799	15591	26	-115.534***
02 October 2019	A	591	10179	22	-92.379***
	B	634	10981	24	-95.998***
	C	1217	18938	31	-124.817***
03 October 2019	A	507	9938	20	-92.269***
	B	708	11251	22	-96.400***
	C	1143	19273	24	-126.879***
04 October 2019	A	512	9969	22	-92.365***
	B	669	11610	20	-98.727***
	C	1221	18970	33	-124.902***
05 October 2019	A	565	9334	7	-88.126***
	B	744	10800	20	-93.584***
	C	1172	16691	23	-116.107***
06 October 2019	A	457	9898	13	-92.768***
	B	577	11011	24	-96.918***
	C	937	16815	20	-119.164***
07 October 2019	A	432	9924	15	-93.264***
	B	551	11523	22	-99.844***
	C	898	20309	25	-133.286***

*** $p < .001$

Table C.3: Sign test results for the transfer time component (for December, 2019).

Departure date	Data collection	S+	S-	Ties	STS
01 December 2019	A	541	9840	18	-91.258***
	B	544	10447	15	-94.451***
	C	746	15605	18	-116.195***
02 December 2019	A	589	9577	15	-89.133***
	B	658	10756	20	-94.509***
	C	758	14922	15	-113.105***
03 December 2019	A	494	8899	14	-86.713***
	B	738	11035	21	-94.891***
	C	804	14057	19	-108.707***
04 December 2019	A	533	9252	26	-88.133***
	B	686	11970	16	-100.294***
	C	800	13693	33	-107.088***
05 December 2019	A	596	9939	20	-91.017***
	B	845	13545	20	-105.862***
	C	955	14639	40	-109.573***
06 December 2019	A	655	11543	22	-98.574***
	B	896	14218	26	-108.355***
	C	938	15389	20	-113.088***
07 December 2019	A	708	8089	25	-78.684***
	B	1055	11767	28	-94.592***
	C	1140	12580	27	-97.659***

*** $p < .001$

Table C.4: Sign test results for the geographical detour factor component (for August, 2019).

Departure date	Data collection	S+	S-	Ties	STS
01 August 2019	A	5797	13736	1999	-56.797 ***
	B	5600	16693	1618	-74.289 ***
	C	6598	15694	2178	-60.916 ***
02 August 2019	A	6001	13697	1980	-54.827 ***
	B	5763	15454	1710	-66.525 ***
	C	6811	15661	1904	-59.030 ***
03 August 2019	A	6094	13632	1562	-53.664 ***
	B	5547	14989	1278	-65.881 ***
	C	6253	15093	1559	-60.499 ***
04 August 2019	A	5695	14999	1874	-64.670 ***
	B	5529	15809	1638	-70.368 ***
	C	6233	16149	1761	-66.274 ***
05 August 2019	A	5520	15194	2002	-67.209 ***
	B	5379	16594	1592	-75.651 ***
	C	6292	16669	2118	-68.475 ***
06 August 2019	A	5185	15344	1793	-70.896 ***
	B	4939	16888	1636	-80.872 ***
	C	5941	16419	1998	-70.065 ***
07 August 2019	A	5342	15002	1695	-67.720 ***
	B	5053	16698	1586	-78.952 ***
	C	5450	15130	1722	-67.470 ***

*** $p < .001$

Table C.5: Sign test results for the geographical detour factor component (for October, 2019).

Departure date	Data collection	S+	S-	Ties	STS
01 October 2019	A	2732	7028	1103	-43.475 ***
	B	3059	8479	1172	-50.449 ***
	C	3654	11641	1121	-64.574 ***
02 October 2019	A	2653	7070	1069	-44.785 ***
	B	2794	7792	1053	-48.567 ***
	C	4288	14405	1493	-73.989 ***
03 October 2019	A	2606	6634	1225	-41.893 ***
	B	2913	7990	1078	-48.613 ***
	C	4558	14412	1470	-71.538 ***
04 October 2019	A	2577	6666	1260	-42.521 ***
	B	2946	8290	1063	-50.406 ***
	C	4446	14400	1378	-72.501 ***
05 October 2019	A	2664	6453	789	-39.672 ***
	B	3030	7669	865	-44.839 ***
	C	4141	12803	942	-66.537 ***
06 October 2019	A	2579	6708	1081	-42.835 ***
	B	2940	7669	1003	-45.903 ***
	C	4005	12547	1220	-66.387 ***
07 October 2019	A	2581	6518	1272	-41.263 ***
	B	2902	8092	1102	-49.489 ***
	C	4363	15360	1509	-78.298 ***

*** $p < .001$

Table C.6: Sign test results for the geographical detour factor component (for December, 2019).

Departure date	Data collection	S+	S-	Ties	STS
01 December 2019	A	2495	6956	948	-45.877 ***
	B	2800	7182	1024	-43.849 ***
	C	3590	11695	1084	-65.549 ***
02 December 2019	A	2356	6826	999	-46.638 ***
	B	2742	7594	1098	-47.715 ***
	C	3245	11289	1161	-66.715 ***
03 December 2019	A	2263	6363	781	-44.134 ***
	B	2820	7980	994	-49.643 ***
	C	3359	10355	1166	-59.732 ***
04 December 2019	A	2430	6554	827	-43.499 ***
	B	2908	8723	1041	-53.910 ***
	C	3053	10325	1148	-62.863 ***
05 December 2019	A	2618	7039	898	-44.978 ***
	B	3077	10186	1147	-61.720 ***
	C	3211	11362	1061	-67.512 ***
06 December 2019	A	2879	8290	1051	-51.191 ***
	B	3191	10697	1252	-63.684 ***
	C	3348	11787	1212	-68.588 ***
07 December 2019	A	2275	5960	587	-40.596 ***
	B	2999	9110	741	-55.525 ***
	C	3045	10103	599	-61.545 ***

*** $p < .001$

Appendix D: Descriptive statistics on the fare, transfer time and geographical detour factor difference distributions in the selected markets

Table D.1: Statistics with regard to the fare difference distribution (for August, 2019); considering only the lowest available virtually interlined (VI) flight itinerary and *indirect* traditional (iTR) flight itinerary for all OD-pairs between which VI is less expensive than iTR and between which VI renders a longer transfer time compared to the iTR alternative.

Departure date	Data collection	Total N	Skewness	Kurtosis	KS statistic	Sig.
01 August 2019	A	19981	4.752	36.696	0.217	.000
	B	22267	4.994	43.976	0.213	.000
	C	22145	4.571	39.417	0.220	.000
02 August 2019	A	20108	5.140	46.000	0.223	.000
	B	21428	4.290	30.962	0.205	.000
	C	21977	4.008	24.806	0.214	.000
03 August 2019	A	19612	3.944	25.280	0.197	.000
	B	20300	3.744	24.965	0.182	.000
	C	20336	4.164	32.795	0.195	.000
04 August 2019	A	21289	4.798	46.423	0.194	.000
	B	21778	4.054	29.185	0.185	.000
	C	22059	3.680	26.152	0.175	.000
05 August 2019	A	21561	4.378	34.842	0.197	.000
	B	22310	3.869	25.780	0.192	.000
	C	22615	3.749	24.066	0.192	.000
06 August 2019	A	21127	5.072	43.506	0.219	.000
	B	22314	4.534	32.237	0.210	.000
	C	21982	3.554	20.911	0.188	.000
07 August 2019	A	20764	5.006	41.270	0.215	.000
	B	22056	5.001	41.893	0.209	.000
	C	19938	3.498	22.511	0.183	.000

*** $p < .001$

Table D.2: Statistics with regard to the transfer time difference distribution (for August, 2019); considering only the lowest available virtually interlined (VI) flight itinerary and *indirect* traditional (iTR) flight itinerary for all OD-pairs between which VI is less expensive than iTR and between which VI renders a longer transfer time compared to the iTR alternative.

Departure date	Data collection	Total N	Skewness	Kurtosis	KS statistic	Sig.
01 August 2019	A	19981	-0.878	0.533	0.079	.000
	B	22267	-0.759	0.163	0.071	.000
	C	22145	-0.868	0.500	0.075	.000
02 August 2019	A	20108	-0.841	0.192	0.084	.000
	B	21428	-0.687	-0.180	0.073	.000
	C	21977	-0.801	0.206	0.077	.000
03 August 2019	A	19612	-0.679	-0.007	0.064	.000
	B	20300	-0.627	-0.197	0.061	.000
	C	20336	-0.859	0.447	0.074	.000
04 August 2019	A	21289	-0.657	-0.089	0.065	.000
	B	21778	-0.589	-0.266	0.059	.000
	C	22059	-0.742	0.134	0.067	.000
05 August 2019	A	21561	-0.722	0.087	0.061	.000
	B	22310	-0.639	-0.126	0.056	.000
	C	22615	-0.768	0.249	0.066	.000
06 August 2019	A	21127	-0.702	0.029	0.064	.000

	B	22314	-0.629	-0.196	0.059	.000
	C	21982	-0.833	0.460	0.068	.000
07 August 2019	A	20764	-0.757	0.223	0.069	.000
	B	22056	-0.731	0.079	0.068	.000
	C	19938	-0.858	0.470	0.074	.000

*** $p < .001$

Table D.3: Statistics with regard to the fare difference distribution (for October, 2019); considering only the lowest available virtually interlined (VI) flight itinerary and *indirect* traditional (iTR) flight itinerary for all OD-pairs between which VI is less expensive than iTR and between which VI renders a longer transfer time compared to the iTR alternative.

Departure date	Data collection	Total N	Skewness	Kurtosis	KS statistic	Sig.
01 October 2019	A	10382	6.043	56.985	0.241	.000
	B	12107	6.888	95.527	0.249	.000
	C	15591	6.017	60.544	0.235	.000
02 October 2019	A	10179	5.600	51.030	0.236	.000
	B	10981	7.077	107.530	0.240	.000
	C	18938	5.444	46.683	0.227	.000
03 October 2019	A	9938	6.782	105.967	0.232	.000
	B	11251	4.501	34.337	0.216	.000
	C	19273	5.032	42.951	0.219	.000
04 October 2019	A	9969	5.957	72.738	0.221	.000
	B	11610	7.120	95.389	0.240	.000
	C	18970	4.826	37.501	0.210	.000
05 October 2019	A	9334	6.334	90.557	0.217	.000
	B	10800	4.550	35.069	0.214	.000
	C	16691	4.753	35.988	0.208	.000
06 October 2019	A	9898	4.911	44.022	0.202	.000
	B	11011	3.760	23.314	0.186	.000
	C	16815	4.368	30.029	0.194	.000
07 October 2019	A	9924	7.730	126.563	0.237	.000
	B	11523	5.132	44.150	0.217	.000
	C	20309	5.049	38.571	0.220	.000

*** $p < .001$

Table D.4: Statistics with regard to the transfer time difference distribution (for October, 2019); considering only the lowest available virtually interlined (VI) flight itinerary and *indirect* traditional (iTR) flight itinerary for all OD-pairs between which VI is less expensive than iTR and between which VI renders a longer transfer time compared to the iTR alternative.

Departure date	Data collection	Total N	Skewness	Kurtosis	KS statistic	Sig.
01 October 2019	A	10382	-0.945	1.047	0.075	.000
	B	12107	-0.915	0.908	0.075	.000
	C	15591	-0.876	0.701	0.071	.000
02 October 2019	A	10179	-0.944	0.828	0.088	.000
	B	10981	-0.915	0.633	0.086	.000
	C	18938	-0.834	0.404	0.080	.000
03 October 2019	A	9938	-1.007	0.977	0.091	.000
	B	11251	-0.934	0.624	0.089	.000
	C	19273	-0.791	0.160	0.079	.000
04 October 2019	A	9969	-0.871	0.628	0.079	.000
	B	11610	-0.844	0.554	0.075	.000
	C	18970	-0.785	0.355	0.067	.000
05 October 2019	A	9334	-0.988	0.850	0.084	.000
	B	10800	-0.939	0.665	0.085	.000
	C	16691	-0.767	0.149	0.072	.000

06 October 2019	A	9898	-0.855	0.662	0.070	.000
	B	11011	-0.776	0.388	0.070	.000
	C	16815	-0.675	0.036	0.063	.000
07 October 2019	A	9924	-0.978	1.057	0.076	.000
	B	11523	-0.976	0.990	0.077	.000
	C	20309	-0.764	0.246	0.061	.000

*** $p < .001$

Table D.5: Statistics with regard to the fare difference distribution (for December, 2019); considering only the lowest available virtually interlined (VI) flight itinerary and *indirect* traditional (iTR) flight itinerary for all OD-pairs between which VI is less expensive than iTR and between which VI renders a longer transfer time compared to the iTR alternative.

Departure date	Data	Total N	Skewness	Kurtosis	KS statistic	Sig.
01 December 2019	A	9840	5.749	52.952	0.247	.000
	B	10447	4.978	38.727	0.237	.000
	C	15605	5.701	51.011	0.224	.000
02 December 2019	A	9577	7.005	85.280	0.267	.000
	B	10756	4.976	35.705	0.248	.000
	C	14922	5.105	35.469	0.232	.000
03 December 2019	A	8899	4.894	31.015	0.265	.000
	B	11035	4.798	29.300	0.258	.000
	C	14057	5.915	56.192	0.257	.000
04 December 2019	A	9252	5.418	42.602	0.263	.000
	B	11970	5.124	35.838	0.245	.000
	C	13693	5.368	40.334	0.237	.000
05 December 2019	A	9939	4.958	33.471	0.248	.000
	B	13545	5.239	41.847	0.237	.000
	C	14639	5.249	38.475	0.238	.000
06 December 2019	A	11543	5.244	40.752	0.253	.000
	B	14218	5.628	49.765	0.247	.000
	C	15389	5.802	48.385	0.245	.000
07 December 2019	A	8089	4.724	30.284	0.236	.000
	B	11767	5.750	46.591	0.234	.000
	C	12580	5.867	48.866	0.231	.000

*** $p < .001$

Table D.6: Statistics with regard to the transfer time difference distribution (for December, 2019); considering only the lowest available virtually interlined (VI) flight itinerary and *indirect* traditional (iTR) flight itinerary for all OD-pairs between which VI is less expensive than iTR and between which VI renders a longer transfer time compared to the iTR alternative.

Departure date	Data	Total N	Skewness	Kurtosis	KS statistic	Sig.
01 December 2019	A	9840	-0.795	0.543	0.070	.000
	B	10447	-0.727	0.495	0.061	.000
	C	15605	-0.632	0.076	0.056	.000
02 December 2019	A	9577	-0.925	0.770	0.085	.000
	B	10756	-0.905	0.731	0.082	.000
	C	14922	-0.797	0.346	0.069	.000
03 December 2019	A	8899	-0.845	0.575	0.082	.000
	B	11035	-0.821	0.378	0.082	.000
	C	14057	-0.744	0.253	0.072	.000
04 December 2019	A	9252	-0.984	0.840	0.089	.000
	B	11970	-0.941	0.651	0.086	.000
	C	13693	-0.913	0.606	0.082	.000
05 December 2019	A	9939	-0.728	0.299	0.077	.000

	B	13545	-0.689	0.072	0.073	.000
	C	14639	-0.697	0.035	0.073	.000
06 December 2019	A	11543	-1.021	0.933	0.083	.000
	B	14218	-0.841	0.322	0.073	.000
	C	15389	-0.778	0.200	0.067	.000
07 December 2019	A	8089	-0.922	0.905	0.083	.000
	B	11767	-0.829	0.450	0.076	.000
	C	12580	-0.866	0.410	0.078	.000

*** $p < .001$

Table D.7: Statistics with regard to the fare difference distribution (for August, 2019); considering only the lowest available virtually interlined flight (VI) itinerary and *indirect* traditional (iTR) flight itinerary for all OD-pairs between which VI is less expensive than iTR and between which VI renders a larger geographical detour factor compared to the iTR alternative.

Departure date	Data collection	Total N	Skewness	Kurtosis	KS statistic	Sig.
01 August 2019	A	13736	4.435	30.410	0.204	.000
	B	16693	5.400	50.684	0.211	.000
	C	15694	4.640	37.962	0.215	.000
02 August 2019	A	13697	5.653	57.761	0.223	.000
	B	15454	3.949	25.068	0.192	.000
	C	15661	4.160	27.488	0.209	.000
03 August 2019	A	13632	4.207	29.542	0.199	.000
	B	14989	4.003	30.259	0.181	.000
	C	15093	4.368	37.555	0.193	.000
04 August 2019	A	14999	4.653	48.065	0.185	.000
	B	15809	4.267	33.517	0.182	.000
	C	16149	3.498	23.764	0.167	.000
05 August 2019	A	15194	4.108	31.348	0.185	.000
	B	16594	3.473	18.739	0.183	.000
	C	16669	3.610	22.254	0.185	.000
06 August 2019	A	15344	5.242	50.843	0.210	.000
	B	16888	4.752	37.809	0.201	.000
	C	16419	3.433	20.187	0.178	.000
07 August 2019	A	15002	4.648	32.890	0.202	.000
	B	16698	5.301	50.330	0.202	.000
	C	15130	3.364	21.881	0.171	.000

*** $p < .001$

Table D.8: Statistics with regard to the GeoDetour difference distribution (for August, 2019); considering only the lowest available virtually interlined flight (VI) itinerary and *indirect* traditional (iTR) flight itinerary for all OD-pairs between which VI is less expensive than iTR and between which VI renders a larger geographical detour factor compared to the iTR alternative.

Departure date	Data collection	Total N	Skewness	Kurtosis	KS statistic	Sig.
01 August 2019	A	13736	-9.126	173.426	0.291	.000
	B	16693	-6.616	71.741	0.282	.000
	C	15694	-10.726	220.113	0.313	.000
02 August 2019	A	13697	-6.992	74.501	0.288	.000
	B	15454	-7.291	89.700	0.283	.000
	C	15661	-9.629	181.855	0.303	.000
03 August 2019	A	13632	-8.763	145.711	0.287	.000
	B	14989	-9.727	161.042	0.300	.000
	C	15093	-6.988	86.766	0.285	.000
04 August 2019	A	14999	-7.135	87.405	0.280	.000
	B	15809	-10.422	200.151	0.299	.000
	C	16149	-9.214	148.310	0.307	.000

05 August 2019	A	15194	-7.685	96.797	0.291	.000
	B	16594	-8.849	132.206	0.300	.000
	C	16669	-8.561	127.743	0.304	.000
06 August 2019	A	15344	-8.194	132.937	0.286	.000
	B	16888	-8.046	115.429	0.291	.000
	C	16419	-9.441	166.460	0.301	.000
07 August 2019	A	15002	-9.053	165.532	0.292	.000
	B	16698	-9.213	157.997	0.295	.000
	C	15130	-8.564	118.320	0.313	.000

*** $p < .001$

Table D.9: Statistics with regard to the fare difference distribution (for October, 2019); considering only the lowest available virtually interlined flight (VI) itinerary and *indirect* traditional (iTR) flight itinerary for all OD-pairs between which VI is less expensive than iTR and between which VI renders a larger geographical detour factor compared to the iTR alternative.

Departure date	Data collection	Total N	Skewness	Kurtosis	KS statistic	Sig.
01 October 2019	A	7028	6.246	60.501	0.243	.000
	B	8479	7.508	120.560	0.247	.000
	C	11641	6.461	71.161	0.233	.000
02 October 2019	A	7070	5.613	51.898	0.232	.000
	B	7792	7.903	133.907	0.243	.000
	C	14405	5.738	53.640	0.222	.000
03 October 2019	A	6634	5.138	43.875	0.223	.000
	B	7990	4.682	37.955	0.215	.000
	C	14412	5.520	53.491	0.218	.000
04 October 2019	A	6666	6.623	97.926	0.214	.000
	B	8290	7.301	103.611	0.230	.000
	C	14400	5.163	45.241	0.207	.000
05 October 2019	A	6453	5.453	58.509	0.208	.000
	B	7669	4.265	28.578	0.209	.000
	C	12803	4.815	36.993	0.203	.000
06 October 2019	A	6708	4.600	37.732	0.194	.000
	B	7669	3.841	24.331	0.187	.000
	C	12547	4.548	33.430	0.191	.000
07 October 2019	A	6518	8.652	156.190	0.239	.000
	B	8092	5.522	51.677	0.218	.000
	C	15360	5.227	42.582	0.215	.000

*** $p < .001$

Table D.10: Statistics with regard to the GeoDetour difference distribution (for October, 2019); considering only the lowest available virtually interlined flight (VI) itinerary and *indirect* traditional (iTR) flight itinerary for all OD-pairs between which VI is less expensive than iTR and between which VI renders a larger geographical detour factor compared to the iTR alternative.

Departure date	Data collection	Total N	Skewness	Kurtosis	KS statistic	Sig.
01 October 2019	A	7028	-10.923	216.592	0.299	.000
	B	8479	-9.236	145.860	0.297	.000
	C	11641	-6.099	59.697	0.278	.000
02 October 2019	A	7070	-10.616	204.849	0.305	.000
	B	7792	-9.099	134.883	0.300	.000
	C	14405	-7.317	90.724	0.282	.000
03 October 2019	A	6634	-6.503	75.347	0.271	.000
	B	7990	-24.972	1186.569	0.334	.000
	C	14412	-13.661	387.412	0.303	.000
04 October 2019	A	6666	-8.989	143.624	0.286	.000
	B	8290	-10.587	194.090	0.299	.000

	C	14400	-8.865	151.502	0.288	.000
05 October 2019	A	6453	-6.524	80.005	0.271	.000
	B	7669	-6.042	61.340	0.270	.000
	C	12803	-12.176	316.848	0.297	.000
06 October 2019	A	6708	-5.822	68.602	0.262	.000
	B	7669	-7.788	106.521	0.288	.000
	C	12547	-6.591	75.700	0.281	.000
07 October 2019	A	6518	-8.031	119.483	0.281	.000
	B	8092	-8.630	134.797	0.287	.000
	C	15360	-7.725	123.932	0.273	.000

*** $p < .001$

Table D.11: Statistics with regard to the fare difference distribution (for December, 2019); considering only the lowest available virtually interlined flight (VI) itinerary and *indirect* traditional (iTR) flight itinerary for all OD-pairs between which VI is less expensive than iTR and between which VI renders a larger geographical detour factor compared to the iTR alternative.

Departure date	Data	Total N	Skewness	Kurtosis	KS statistic	Sig.
01 December 2019	A	6956	6.321	65.042	0.247	.000
	B	7182	5.428	49.044	0.233	.000
	C	11695	6.336	66.265	0.219	.000
02 December 2019	A	6826	7.055	88.510	0.259	.000
	B	7594	5.311	41.768	0.245	.000
	C	11289	5.426	41.570	0.224	.000
03 December 2019	A	6363	5.172	34.876	0.266	.000
	B	7980	4.961	31.623	0.255	.000
	C	10355	6.745	77.765	0.254	.000
04 December 2019	A	6554	5.548	43.233	0.258	.000
	B	8723	5.456	41.515	0.243	.000
	C	10325	5.279	37.894	0.224	.000
05 December 2019	A	7039	4.999	33.944	0.249	.000
	B	10186	5.623	49.743	0.236	.000
	C	11362	5.655	46.410	0.234	.000
06 December 2019	A	8290	5.726	49.204	0.256	.000
	B	10697	5.605	45.243	0.238	.000
	C	11787	6.153	56.010	0.239	.000
07 December 2019	A	5960	5.041	36.096	0.232	.000
	B	9110	6.313	58.193	0.230	.000
	C	10103	6.512	60.523	0.231	.000

*** $p < .001$

Table D.12: Statistics with regard to the GeoDetour difference distribution (for December, 2019); considering only the lowest available virtually interlined flight (VI) itinerary and *indirect* traditional (iTR) flight itinerary for all OD-pairs between which VI is less expensive than iTR and between which VI renders a larger geographical detour factor compared to the iTR alternative.

Departure date	Data	Total N	Skewness	Kurtosis	KS statistic	Sig.
01 December 2019	A	6956	-8.794	133.829	0.304	.000
	B	7182	-11.534	239.867	0.314	.000
	C	11695	-14.981	479.996	0.302	.000
02 December 2019	A	6826	-19.894	711.092	0.337	.000
	B	7594	-26.790	1107.915	0.348	.000
	C	11289	-21.491	821.581	0.320	.000
03 December 2019	A	6363	-12.889	283.418	0.324	.000
	B	7980	-25.402	994.699	0.343	.000
	C	10355	-22.349	786.535	0.338	.000

04 December 2019	A	6554	-26.590	1036.381	0.359	.000
	B	8723	-27.913	1260.374	0.334	.000
	C	10325	-25.254	1024.223	0.328	.000
05 December 2019	A	7039	-21.751	824.093	0.345	.000
	B	10186	-21.769	832.276	0.329	.000
	C	11362	-18.200	648.260	0.315	.000
06 December 2019	A	8290	-17.542	598.472	0.323	.000
	B	10697	-22.853	884.847	0.323	.000
	C	11787	-17.644	575.439	0.316	.000
07 December 2019	A	5960	-21.384	878.145	0.327	.000
	B	9110	-18.295	753.672	0.297	.000
	C	10103	-16.499	573.132	0.306	.000

*** $p < .001$

Appendix E: Correlation tests

Table E.1: Correlation test of the iTR-VI fare > 0 distribution and the iTR-VI transfer time < 0 distribution (for August, 2019).

Departure date	Data collection	Total N	Kendall's tau-b		Spearman's rho	
			Corr. Coeff.	Sig.	Corr. Coeff.	Sig.
01 August 2019	A	19981	-0.114**	.000	-0.170**	.000
	B	22267	-0.122**	.000	-0.182**	.000
	C	22145	-0.100**	.000	-0.149**	.000
02 August 2019	A	20108	-0.120**	.000	-0.180**	.000
	B	21428	-0.126**	.000	-0.189**	.000
	C	21977	-0.108**	.000	-0.161**	.000
03 August 2019	A	19612	-0.126**	.000	-0.188**	.000
	B	20300	-0.131**	.000	-0.195**	.000
	C	20336	-0.104**	.000	-0.155**	.000
04 August 2019	A	21289	-0.143**	.000	-0.213**	.000
	B	21778	-0.161**	.000	-0.240**	.000
	C	22059	-0.123**	.000	-0.183**	.000
05 August 2019	A	21561	-0.155**	.000	-0.231**	.000
	B	22310	-0.151**	.000	-0.226**	.000
	C	22615	-0.130**	.000	-0.194**	.000
06 August 2019	A	21127	-0.136**	.000	-0.203**	.000
	B	22314	-0.131**	.000	-0.196**	.000
	C	21982	-0.113**	.000	-0.168**	.000
07 August 2019	A	20764	-0.125**	.000	-0.186**	.000
	B	22056	-0.113**	.000	-0.168**	.000
	C	19938	-0.087**	.000	-0.130**	.000

** $p < .01$

Table E.2: Correlation test of the iTR-VI fare > 0 distribution and the iTR-VI transfer time < 0 distribution (for October, 2019).

Departure date	Data collection	Total N	Kendall's tau-b		Spearman's rho	
			Corr. Coeff.	Sig.	Corr. Coeff.	Sig.
01 October 2019	A	10382	-0.050**	.000	-0.075**	.000
	B	12107	-0.074**	.000	-0.111**	.000
	C	15591	-0.088**	.000	-0.131**	.000
02 October 2019	A	10179	-0.047**	.000	-0.071**	.000
	B	10981	-0.054**	.000	-0.080**	.000
	C	18938	-0.085**	.000	-0.126**	.000
03 October 2019	A	9938	-0.063**	.000	-0.093**	.000
	B	11251	-0.059**	.000	-0.088**	.000
	C	19273	-0.102**	.000	-0.152**	.000
04 October 2019	A	9969	-0.079**	.000	-0.117**	.000
	B	11610	-0.085**	.000	-0.126**	.000
	C	18970	-0.104**	.000	-0.155**	.000
05 October 2019	A	9334	-0.067**	.000	-0.101**	.000
	B	10800	-0.062**	.000	-0.092**	.000
	C	16691	-0.088**	.000	-0.131**	.000
06 October 2019	A	9898	-0.093**	.000	-0.138**	.000
	B	11011	-0.095**	.000	-0.142**	.000
	C	16815	-0.127**	.000	-0.189**	.000
07 October 2019	A	9924	-0.072**	.000	-0.108**	.000
	B	11523	-0.070**	.000	-0.104**	.000
	C	20309	-0.103**	.000	-0.153**	.000

** $p < .01$

Table E.3: Correlation test of the iTR-VI fare > 0 distribution and the iTR-VI transfer time < 0 distribution (for December, 2019).

Departure date	Data collection	Total N	Kendall's tau-b		Spearman's rho	
			Corr. Coeff.	Sig.	Corr. Coeff.	Sig.
01 December 2019	A	9840	-0.069**	.000	-0.103**	.000
	B	10447	-0.068**	.000	-0.101**	.000
	C	15605	-0.099**	.000	-0.147**	.000
02 December 2019	A	9577	-0.087**	.000	-0.129**	.000
	B	10756	-0.101**	.000	-0.151**	.000
	C	14922	-0.095**	.000	-0.142**	.000
03 December 2019	A	8899	-0.073**	.000	-0.108**	.000
	B	11035	-0.093**	.000	-0.140**	.000
	C	14057	-0.098**	.000	-0.146**	.000
04 December 2019	A	9252	-0.073**	.000	-0.108**	.000
	B	11970	-0.085**	.000	-0.126**	.000
	C	13693	-0.068**	.000	-0.101**	.000
05 December 2019	A	9939	-0.078**	.000	-0.117**	.000
	B	13545	-0.084**	.000	-0.126**	.000
	C	14639	-0.069**	.000	-0.103**	.000
06 December 2019	A	11543	-0.074**	.000	-0.109**	.000
	B	14218	-0.073**	.000	-0.110**	.000
	C	15389	-0.071**	.000	-0.106**	.000
07 December 2019	A	8089	-0.073**	.000	-0.109**	.000
	B	11767	-0.078**	.000	-0.116**	.000
	C	12580	-0.053**	.000	-0.079**	.000

** $p < .01$

Table E.4: Correlation test of the iTR-VI fare > 0 distribution and the iTR-VI geographical detour factor < 0 distribution (for August, 2019).

Departure date	Data collection	Total N	Kendall's tau-b		Spearman's rho	
			Corr. Coeff.	Sig.	Corr. Coeff.	Sig.
01 August 2019	A	13736	0.042**	.000	0.064**	.000
	B	16693	0.048**	.000	0.072**	.000
	C	15694	0.028**	.000	0.041**	.000
02 August 2019	A	13697	0.061**	.000	0.091**	.000
	B	15454	0.072**	.000	0.108**	.000
	C	15661	0.023**	.000	0.034**	.000
03 August 2019	A	13632	0.066**	.000	0.098**	.000
	B	14989	0.074**	.000	0.111**	.000
	C	15093	0.038**	.000	0.057**	0.000
04 August 2019	A	14999	0.065**	.000	0.098**	.000
	B	15809	0.052**	.000	0.077**	.000
	C	16149	0.007	.203	0.010	0.206
05 August 2019	A	15194	0.049**	.000	0.074**	.000
	B	16594	0.053**	.000	0.078**	.000
	C	16669	-0.008	0.126	-0.012	0.121
06 August 2019	A	15344	0.039**	.000	0.059**	.000
	B	16888	0.057**	.000	0.085**	.000
	C	16419	-0.001	0.898	-0.001	0.897
07 August 2019	A	15002	0.055**	.000	0.082**	.000
	B	16698	0.058**	.000	0.087**	.000
	C	15130	-0.030**	.000	-0.044**	.000

** $p < .01$

Table E.5: Correlation test of the iTR-VI fare > 0 distribution and the iTR-VI geographical detour factor < 0 distribution (for October, 2019).

Departure date	Data collection	Total N	Kendall's tau-b		Spearman's rho	
			Corr. Coeff.	Sig.	Corr. Coeff.	Sig.
01 October 2019	A	7028	0.067**	.000	0.100**	.000
	B	8479	0.079**	.000	0.117**	.000
	C	11641	0.101**	.000	0.151**	.000
02 October 2019	A	7070	0.064**	.000	0.095**	.000
	B	7792	0.078**	.000	0.115**	.000
	C	14405	0.078**	.000	0.116**	.000
03 October 2019	A	6634	0.045**	.000	0.067**	.000
	B	7990	0.042**	.000	0.062**	.000
	C	14412	0.062**	.000	0.093**	.000
04 October 2019	A	6666	0.069**	.000	0.103**	.000
	B	8290	0.076**	.000	0.113**	.000
	C	14400	0.084**	.000	0.126**	.000
05 October 2019	A	6453	0.086**	.000	0.127**	.000
	B	7669	0.061**	.000	0.091**	.000
	C	12803	0.081**	.000	0.120**	.000
06 October 2019	A	6708	0.079**	.000	0.116**	.000
	B	7669	0.060**	.000	0.089**	.000
	C	12547	0.073**	.000	0.109**	.000
07 October 2019	A	6518	0.075**	.000	0.111**	.000
	B	8092	0.061**	.000	0.091**	.000
	C	15360	0.087**	.000	0.130**	.000

** $p < .01$

Table E.6: Correlation test o of the iTR-VI fare > 0 distribution and the iTR-VI geographical detour factor < 0 distribution (for December, 2019).

Departure date	Data collection	Total N	Kendall's tau-b		Spearman's rho	
			Corr. Coeff.	Sig.	Corr. Coeff.	Sig.
01 December 2019	A	6956	0.088**	.000	0.132**	.000
	B	7182	0.099**	.000	0.148**	.000
	C	11695	0.028**	.000	0.041**	.000
02 December 2019	A	6826	0.090**	.000	0.133**	.000
	B	7594	0.113**	.000	0.168**	.000
	C	11289	0.042**	.000	0.062**	.000
03 December 2019	A	6363	0.130**	.000	0.194**	.000
	B	7980	0.069**	.000	0.103**	.000
	C	10355	0.048**	.000	0.072**	.000
04 December 2019	A	6554	0.116**	.000	0.172**	.000
	B	8723	0.094**	.000	0.139**	.000
	C	10325	0.064**	.000	0.096**	.000
05 December 2019	A	7039	0.117**	.000	0.174**	.000
	B	10186	0.102**	.000	0.151**	.000
	C	11362	0.082**	.000	0.121**	.000
06 December 2019	A	8290	0.117**	.000	0.173**	.000
	B	10697	0.082**	.000	0.122**	.000
	C	11787	0.069**	.000	0.102**	.000
07 December 2019	A	5960	0.112**	.000	0.166**	.000
	B	9110	0.090**	.000	0.133**	.000
	C	10103	0.071**	.000	0.105**	.000

** $p < .01$
